# Psychoanalytic psychotherapies and the free energy principle

**DOI:** 10.3389/fnhum.2022.929940

**Published:** 2022-08-09

**Authors:** Thomas Rabeyron

**Affiliations:** ^1^Department of Psychology (Interpsy), University of Lorraine, Nancy, France; ^2^Department of Psychology (KPU), University of Edinburgh, Edinburgh, United Kingdom; ^3^Institut Universitaire de France, Paris, France

**Keywords:** psychoanalysis, free energy, entropy, free association, dream, psychoanalytic therapy, transference, symbolization

## Abstract

In this paper I propose a model of the fundamental components of psychoanalytic psychotherapies that I try to explicate with contemporary theories of the Bayesian brain and the Free Energy Principle (FEP). I first show that psychoanalytic therapies require a setting (made up of several envelopes), a particular psychic state and specific processes (transference, free association, dreaming, play, reflexivity and narrativity) in order to induce psychic transformations. I then analyze how these processes of transformations operate and how they can be enlightened by the FEP. I first underline the fact that psychoanalytic therapies imply non-linear processes taking time to unfold and require a setting containing high entropy processes. More precisely, these processes are characterized by an interplay between extension and reduction of free energy. This interplay also favors the emergence of new orders of subjective experience, which occur following states of disorder, according to a certain energetic threshold allowing the modification and improvement of mental functioning. These high entropy states are also characterized by random functioning and psychic malleability which favors the exploration of subjective experience in an original manner. Overall, the approach proposed in this paper support the dialogue between psychoanalysis and other fields of research while underlining how psychoanalytical theoretical and conceptual constructs can also be useful to other disciplines, in particular the neurosciences of subjectivity.

## Psychoanalytic psychotherapies and the free energy principle

In this paper I propose a model of psychoanalytic psychotherapies associated with the free energy principle (FEP). The FEP assumes that any living organism must reduce its internal free energy, which is generated by the tendency of matter toward disorganization due to the second law of thermodynamics (Friston, 2013). This degree of organization, or disorganization, can be measured in physics by the notion of entropy. Living organisms must therefore manage to fight against the increase of entropy (and thus of disorganization) induce by the environment and maintain a sufficient level of organization by reducing free energy. This principle is considered by Karl Friston as being as fundamental as natural selection or gravity (Badcock et al., 2019; Holmes, 2021).

The psychic apparatus can be considered as a hierarchically organized subsystem having the same function of entropy and free energy reduction by developing a set of predictions about the environment based on Bayesian statistics (Friston et al., 2006; Hirsh et al., 2012; Badcock et al., 2019). As summarized by Cieri et al. (2021): “According to the free energy principle, the brain is an open, adaptive, complex system far from equilibrium and as with any adaptive self-organizing biological system in non-equilibrium steady-state with environment, it must reduce its free energy to resist a natural tendency to disorder” (p. 2). Consequently, it might be relevant to try to understand the psychic apparatus from the FEP (Friston, 2013) knowing that, when it is overwhelmed in its ability to regulate free energy, it can rely on a second and external system to regain its internal homeostasis (Friston and Frith, 2015). This process is present from the start of the psychic life and corresponds to complex interrelations between the infant and the attachment figures (Beebe and Lachmann, 2020). Concretely, the states of disorganization of the baby can be regulated by parental care because the psychic apparatus of the baby, as well as its biological functioning in a more general way, is not sufficiently mature to be autonomous. As Winnicott said “there is no such thing as a baby.”

Psychotherapy consists of a relationship whose final aim is to help the patient to find a form of psychic equilibrium and the psychotherapeutic process may also be understood from the point of view of the FEP^1^ (Chekroud, 2015; Holmes, 2020, 2021). Psychotherapies correspond to highly complex processes and we still have difficulties in grasping precisely the principles of their action (Cuijpers et al., 2019). They are usually evaluated depending on their theoretical frame of reference (psychoanalytic, cognitive-behavioral therapy, humanistic, systemic, etc.) with the aim of demonstrating their effectiveness for different forms of mental disorders. However, such an approach has led to limited progress, notably because we have great difficulty in discriminating the influence of common and specific factors of therapeutic effectiveness (Cuijpers et al., 2019). In addition, there is often a gap between what a clinician says about what he or she is doing -particularly on the basis of theoretical orientation- and what is actually done in clinical practice (Ablon and Jones, 1998). Consequently, the most important question is not if psychotherapies work, but *how* they work. One of the ways to go beyond these controversies could be to rely on more fundamental principles such as the FEP to better understand the transformations induced by psychotherapies and thus promote a “meta-perspective of psychotherapy integration” (Holmes, 2020, p. 165). In this regard, the main processes involved in psychoanalytical psychotherapies^2^ will analyzed in this paper within such a meta-perspective^3^.

## Setting and psychic state during psychoanalytical therapies

**Figure 1:**
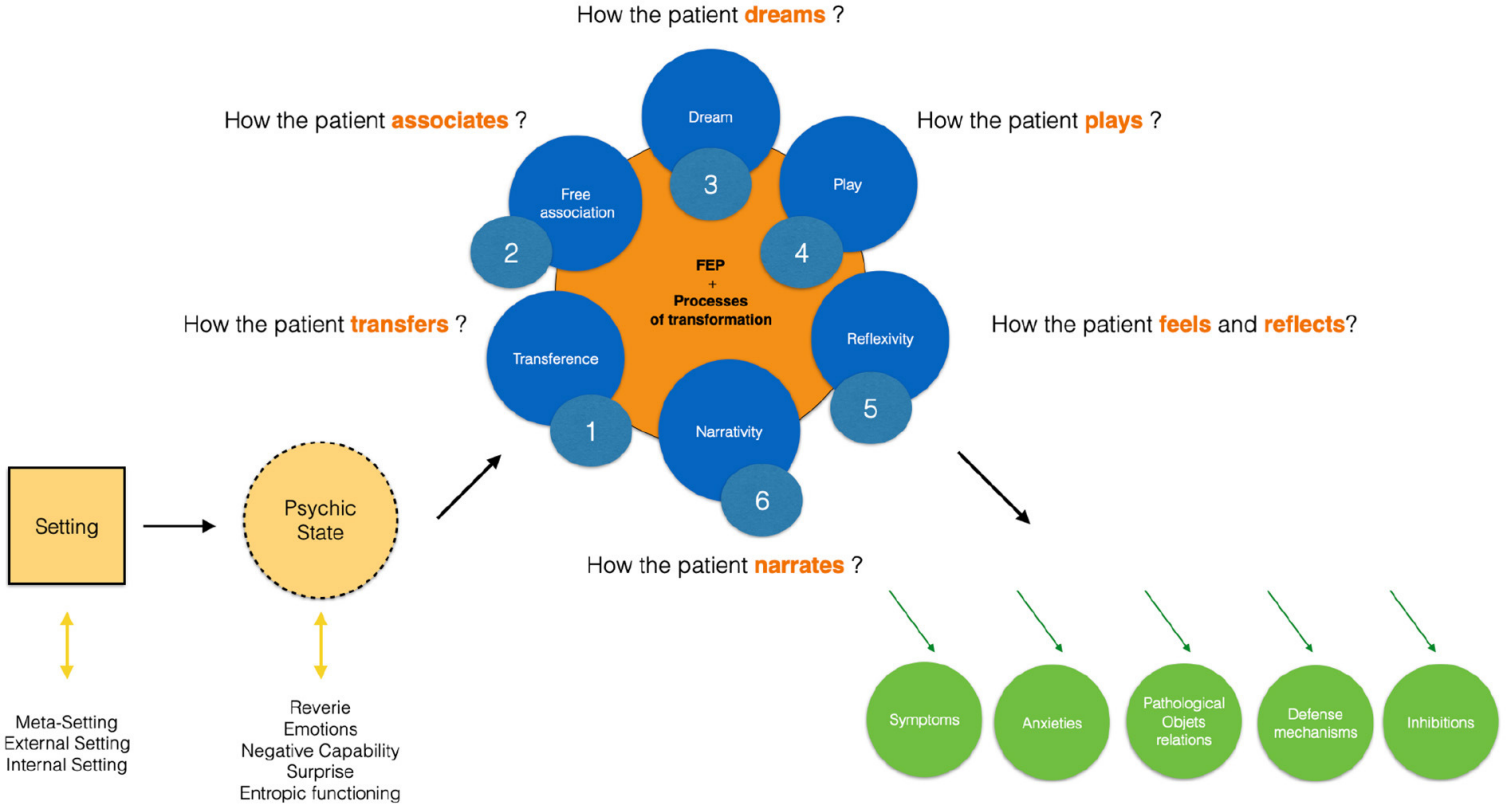
Main components of psychoanalytical therapies.

I am going first to propose a synthetic model of psychoanalytical psychotherapies identifying several components characteristic of this approach (Figure 1)^4^. I propose to distinguish more precisely: (1) the setting (internal, external and meta), (2) the psychic state of the patient and the therapist, (3) the main processes (transference, associativity, dream, play, reflexivity and narrativity), and (4) specific indicators of the therapy effectiveness (symptoms, object relations, defense mechanisms, anxieties and inhibitions). Each of these components will be described before determining to what extent they can be addressed according to the FEP^5^.

### Setting

Psychoanalytic psychotherapies are usually characterized by paying particular attention to the setting (Bleger, 1979). This setting is made up of three complementary envelopes that fit together like Russian dolls: (1) the meta-setting, (2) the external setting, and (3) the internal setting. The meta-setting corresponds to the place where the therapy takes place (for example, a hospital) and more broadly the anthropological and sociological space where the therapy is conducted (for example, in a specific country at a given time). Inside this meta-setting, the external setting corresponds to the space where the therapy takes place (most of the time a consultation room) and all the elements that characterize this therapeutic space: arrangement of the furniture, decoration, light, noise, etc. These elements create an atmosphere that in itself has an impact on the nature of the processes that will take place during the therapy (Rebollar and Rabeyron, 2016). This setting must also be sufficiently “tough” or “structured” in the sense that it has to allow the patient to feel safe to explore her or his psychic life in complete security. For example, the patient has to be assured that what is said during the sessions is strictly confidential and cannot be overheard outside the consultation room. The third dimension of the setting corresponds to the clinician's internal setting -also calls “clinical positioning” (Roussillon et al., 2014)- which is based on implicit principles and explicit rules that guide how the clinician interact with the patient and considers the nature of the therapeutic process.

From the point of view of the FEP, the setting appears as a container whose structure allows, as we will see, a double tendency of extension and reduction of free energy during the therapy. In the same manner that the walls of a nuclear reactor contain the reactions induced by nuclear fission, the setting has to be resistant enough to contain the psychological reactions that occur within it^6^. This is all the more important given that some patients, particularly suffering from borderline personality disorder, regularly seek to “test” the quality of the setting. These patients will also tend to “dialogue” through the setting, i.e., project certain psychological processes onto the setting (Roussillon, 2014). Thus, just as the FEP implies in a living organism the constitution of an envelope to differentiate the internal world from the external world^7^ (Kirchhoff et al., 2018), the setting of a therapy operates as an envelope that contains free energy. Understanding the processes and effects of psychoanalytic psychotherapies therefore implies taking into account the properties of this envelope represented by the different layers of the setting. For example, when this setting is too porous, unstable or even implodes, the therapeutic process will lose its strength, or even collapse, like a cell whose membrane no longer holds its role of delimitation between the internal and the external worlds^8^.

### Psychic state

Psychoanalytic psychotherapies also rely on the implementation of a specific psychic state shared by the patient and the therapist. This state is favored by free association^9^, which encourages the patient to let go of the usual cognitive psychological functioning (Barratt, 2017; Rabeyron and Massicotte, 2020). The patient is invited to express spontaneously, and without restraint, everything that comes to mind. In this way, the attention is focused on internal experience in a very specific manner leading to the expression of different forms of affects and representations. At the same time, the clinician is also in a specific state of listening called by Freud (1919) “floating attention” which implies freely associating in reaction to the patient's free associations. The result is a state of shared “reverie” for both the patient and the clinician, which aims to catalyze the processes of symbolization and transformation.

From the perspective of the FEP, this reverie state appears to both promote the increase of emotional and primary processes^10^ (which increase free energy) and the decrease of cognitive and secondary processes (which reduce free energy). This psychic state, which is also partly found in certain meditation practices^11^, also leads to a state of receptivity -that Bion (1965) calls “negative capability”- which allows the patient and the clinician to be surprised by what emerges in the analytical setting (Reik, 1935; Scarfone, 2018). From a Bayesian perspective, surprise is seen as the effect of increased free energy due to a discrepancy between the external world and the a priori predictions created by the brain (Friston et al., 2006). The other forms of emotions and affects are also catalyzed in this psychic state and correspond to an increase of bottom-up processes. In this sense, the setting and a certain “disposition of mind” favors entropic functioning and a form of disorganization of the usual psychological activity during psychotherapy.

## Main psychological processes of psychoanalytic psychotherapies and FEP

The setting and the psychic state which characterize psychoanalytic therapies are necessary for the emergence of more specific processes. In this regard, six fundamental processes can be distinguished: transference, free association, dreaming, play, reflexivity, and narrativity. I will briefly outline each of these notions through the prism of the FEP and I will hypothesize that they might help to understand how psychoanalytical psychotherapies lead to a “regulation,” or even a “handling,” of free energy in order to induce psychological transformations. These notions will be presented from the most primary to the most secondary level, knowing, however, that they are deeply interconnected and that distinguishing them in this way is necessarily artificial.

**(1) Therapeutic relationship and transference**: the therapeutic relationship is like a channel that allows two psyches—those of the patient and the clinician—to become sufficiently attuned to each other to succeed in inducing transformational processes. There is indeed a “we-mode” characterizing the psychic functioning and allowing two people to find, through complex intersubjective exchanges, a new state of equilibrium (Frith, 2012; Friston and Frith, 2015). This we-mode is probably the core of the therapeutic process, as shown by the importance of the therapeutic alliance in therapeutic effectiveness research (Horvath and Luborsky, 1993; Connolly, 2022). However, it is a necessary but not sufficient parameter for more complex processes to emerge. In this respect, the clinician seems to represent an “auxiliary system” for the self of the patient. The therapeutic relationship thus appears to be a privileged tool for reducing free energy insofar as the clinician's psyche helps to regulate the patient's psyche, in the same way, metaphorically, that, in Newtonian physics, one object transmits its energy to another object. Therapy can thus be conceptualized, at its most basic level, as an energy transference. For example, a patient in a state of agitation necessarily transfers something of this state to the clinician whose containment allows, at a strictly economic level, to reduce the patient's free energy as unbound and unelaborated psychic energy. The passage through the other is thus both a vector of regulation and symbolization^12^ which implicitly rests on a reduction of free energy according to a basic narcissistic identification (Roussillon, 2014). We are then dealing with a very archaic and primary dimension of care relying mainly on the containing, regulating and affective function of the therapist as a mother can be with the infant (Bion, 1962).

This process of intersubjective regulation involves two particular steps. The first is a synchronization which implies that the two psyches “agree” sufficiently to exchange information. This tuning can be analyzed from the point of view of Bayesian systems by taking as a model the synchronization of two birds (Friston and Frith, 2015) and is also supported by the observation of early relationships between babies and their attachment figures (Stern, 1989). From this point of view, psychotherapy can be considered as the meeting of two Bayesian brains which, in order to improve their respective predictions, must agree sufficiently to progressively refine their generative models^13^. This permits a second step of “mixing” of the psyches that goes beyond a simple tuning as noted by Connolly (2022): “the emergence of this synchrony involves a dissolution of boundaries between the two participants where the sensory input from one becomes the prior of the other, as if it emerged from within” (p. 9). Holmes (2020) also explains that “if two inter-acting agents, drawing on the mirror neuron system, mutually assume the other is like themselves, the energetic boundaries between them are temporarily dismantled. Together they build a shared brain or niche” (p. 57). This shared brain evokes what is called the “analytical field” in psychoanalysis, which implies a state of psychic regression for the therapist and the patient in order to produce enduring processes of transformation (Rabeyron, 2020a,b). Such a psychological functioning also seems to catalyzes the transferential processes and induces a greater psychic malleability.

These processes are of great complexity and fall under different registers beyond the topic of therapeutic relationship, which leads naturally to the notion of transference and the unconscious dimension of the therapeutic relationship. Transference is classically considered as the way in which the patient spontaneously tends to unconsciously transfer relational patterns onto the figure of the therapist and the therapeutic relationship (Freud, 1919). This is a central component of psychoanalytic therapies and implies that the clinician accepts to be “taken for another” and be “impregnated” by the patient's psychic life. For example, the patient will behave toward the therapist in the same way that he or she has behaved toward one of the parents (or, conversely, as one of the parents used to behave toward the patient). These relational patterns are so natural for the patients that they tend to interact with the others, and thus with the therapist, according to the same principles, which can lead to pathological relation patterns taking in particular the form of the repetition compulsion (Freud, 1920). In return, the clinician will be particularly attentive to the countertransference, i.e., the way in which the patient induces, often unconsciously at first, specific relational patterns and ways of thinking that inform the clinician about the patient's psychic life. How can such a conception of transference can be interpreted from the point of view of the FEP?

First of all, we could consider that the patient express, within the setting of the therapy, relationship patterns which have previously led to a disorganization of the subjective experience. The analysis of transference thus offers the possibility for the patient to play and integrate, in a reflexive manner, specific psychological functioning and pathological relational patterns. This reflexive loop is concomitant with the internalization of therapeutic intersubjectivity which leads to the integration of new styles of emotional regulation. In this sense, transference allows, usually after a long period of therapy, a progressive “updating” of the relational dimension of the generative model, whose primary function is the reduction of free energy. As Freud (1919) already pointed out, the most efficient way to do this “updating” consists in re-playing, in an unconscious way, these relational patterns in the therapeutic setting. The latter then becomes an “attractor” that will favor the emergence of a “transference neurosis” which corresponds to the replay of pathological relational patterns, in the here and now, on the figure of the therapist. This process is what Freud (1919) has called the *agieren* and might be considered, in more contemporary terms, as a process of enaction (Sapisochin, 2013).

**(2) Free association** is usually considered the most fundamental rule of analytical practices (Barratt, 2017) and requires a form of “letting go” of mental associations without trying to control them. The patient often goes through several steps and, in particular, through a phase of “focal association” in which an event about reality is reported and from which mental associations will be produced. Gradually, as the therapy progresses, the patient learns -or rather unlearns- to not control his or her thoughts, which leads to a more “unbridled” or “extended” form of free association. This allows, as in certain meditation practices, to observe—with attention and without intention—the internal associative flow of consciousness (Rabeyron and Finkel, 2020). Thus, as explained by Holmes (2020), “The job of free association is to slow things down so they can be identified and subjected to rational top-down scrutiny” (2020, p. 108). This state seems to help the patient to express and explore his internal experience, and to register and translate previous experiences in an original manner, which favors reflexivity and transformational processes (Rabeyron and Massicotte, 2020)^14^. The withdrawing from the external world favored by free association results in an increase of free energy and a more chaotic and stochastic experience. Free association then participates in a form of disorganization of the experience which necessarily induces an increase of free energy, which we shall return to in more details later.

**(3) Dreaming and reverie** also appear as fundamental components of psychoanalytical therapies. It concerns both the nocturnal dream activity that the patient may report during the sessions -considering that dreams represent the “royal road to the unconscious” (Freud, 1900)- but it also concerns the state of reverie into which the patient enters during the sessions themselves. In this respect, it is worth remembering that Freud (1940) initially proposed a distinction between free energy and bound energy: “we seem to recognize that nervous or psychic energy occurs in two forms, one freely mobile and another, by comparison, bound” (p. 141). The first corresponds to primary processes in which the energy is considered freer, more mobile, and leads to a rapid discharge in the psychic apparatus. The result is a psychic functioning whose dreaming activity is a typical example and in which a desire might be expressed in a hallucinatory manner. At the opposite, the bound energy corresponds to an energy that is the object of a linking process in the psychic apparatus, notably by cathexis, and whose integration at a higher level of organization induces a more delayed and controlled discharge. There are thus for Freud two levels of “flow” of energy: the first one corresponds to the most primary and unconscious level in which free energy is freer as expressed in the dreams, whereas the second level implies the “handling” of small quantities of energy by consciousness.

Freud (1901) also considers that dreams allow the unconscious processes to be revealed in a more “reliable” manner than in the usual cognitive state as they express more easily unconscious desires and traumatic experiences. Thus, the clinician will pay particular attention to the dreams reported by the patient and may propose interpretations about their meaning. The therapist also plays a specific role during the narrative of the patient's dream by asking what are the mental associations spontaneously emerging, with the aim to decipher the latent content of the dreams. The dream creates a “virtual reality space” (Hopkins, 2016) that allows the exploration of desires and traumatic experiences that have not been integrated and that can be expressed in complete safety given that the subject does not act-out in reality the content of the dreams. Then, when the patient becomes capable of elaborating the unelaborated, of making the unconscious conscious, notably through this work focusing on dream expression and analysis, he or she is more in contact with the feelings (internal reality) and with the environment (external reality), and thus refines and perfects the generative model. As proposed by Holmes (2020), the “granulosity” of the world has been improved and more “nuances” are accessible to the patient. As a result, the patient builds a model of reality that diminish free energy because the ability to represent, to predict and to act precisely has been enhanced.

But dream activity cannot be reduced to the nocturnal dreams and there is actually a much bigger “dream function” as shown by Bion (1965). The latter supposes that dream processes are always present in the background of psychological activity, even during the usual cognitive states, and have the function of making perceptive, sensory and emotional experiences “thinkable,” integrable and subjectivable. This process of symbolization through dreams would be catalyzed by the states of reverie that we have already evoked. The patient enters in a state that allows him to dream his experience and to “dream undreamt dreams” (Ogden, 2012), this state being catalyzed by the analyst's own dream function that Bion (1965) calls “dream-work-alpha.” Dreaming thus appears to be essential in the integration of free energy as Holmes and Nolte (2019) point out: “From the point of view of the FEP, dreaming elaborates the trauma so that it becomes thinkable (p. 9).” This work of psychic integration passes, just as for the free association, by a state of “letting go” which allows the exploration of new states and new configurations of the subjective experience, which, *in fine*, transforms the psychic apparatus and the generative model.

**(4) Play** is also a central component of psychoanalytic therapies which can be considered as “space of play” for both the clinician and the patient in order to explore new experiences. But the patient has to be able to play as Winnicott (1971) pointed out: “Psychotherapy takes place in the overlap of two areas of playing, that of the patient and that of the therapist. Psychotherapy has to do with two people playing together. The corollary of this is that where playing is not possible then the work done by the therapist is directed toward bringing the patient from a state of not being able to play into a state of being able to play” (p. 39). Play appears more precisely as a “state” or a “capacity” that allows unsymbolized and unplayed experiences to be “brought into play” (Roussillon, 2008). But, these the traumatic components of these experiences induce a potential increase of free energy and a disorganization of the psychic apparatus.

Play then implies the regulation of this free energy in a “transitional space” (Winnicott, 1969), i.e., a space of transition between the internal and the external world for experiences not yet integrated. The play is to be distinguished from symptoms which, on the contrary, are “crystallized” and lead the subject to remain the prisoner of pathological relational patterns and behaviors, which ultimately disturb internal psychological homeostasis. The transitional space also evokes certain cerebral states of “criticality” which corresponds to a transition zone between order and chaos (Cieri et al., 2021): “Critical behavior means that activity of the brain continuously transits between two phases, one in which activity increases and amplifies over time and another in which activity rapidly reduces and dies. Between these two phases, there is a critical zone in which the system increases information processing” (p. 12). The brain alternates between phases of order and disorder, which also characterizes the processes of play and the ability to tolerate a certain degree of disorder to “order” the subjective experience differently. This requires a form of “illusion” corresponding to a particular conjunction between the internal and the external world described by Winnicott (1969) as the “paradox of found-created”: the subject must have the impression that he creates the world where he finds it. This paradox gives the feeling that the representation of the world, as a complex “hallucination,” coincides sufficiently with the world in itself. This is summed up by Frith (2007) “our perception of the world is a fantasy that coincides with reality” (p. 111) and, in terms of the FEP, a sufficient “co-incidence” between the generative model and the environment seems to be necessary to “update” the generative model.

Playing therefore represents an activity allowing the transformation of psychic reality through the exploration of new states of mind. This work of exploration aims in particular at integrating the non-integrated and “playing the non-played,” which underlines the extent to which the *nachträglichkeit* (afterwardness) is a fundamental aspect of psychological functioning. The psychic apparatus is indeed constituted in such a way that there is always a “gap” between what the subject can integrate from his experience (internal and external) and what he perceives or feels of it. In this sense, the play makes it possible to rework non-integrated experiences in the *nachträglichkeit*, and in particular traumatic experiences that could jeopardize the structure of the psychic apparatus or the generative model because of an uncontrolled influx of free energy^15^. The play thus appears to be a complex activity of “putting back into play” experiences that have induced too much entropy to be integrated under “good enough” conditions. This is probably the reason why play induces a form of disorder but sufficiently controlled to regulate free energy. Otherwise, the play is “overflowed” and loses its fundamental characteristic of being a play^16^.

**(5) Reflexivity** appears as an additional level of psychological functioning which corresponds to the ability to represent the experience according to a “meta” layer (Roussillon, 1991). The human species has developed a degree of reflexivity whose depth is unique in the animal kingdom. This ability allows us to “feel our feelings” and to “think our thoughts,” in an extremely complex manner, which permits us to project ourselves in space and time, thus offering new capacities of adaptation and control of the environment. But this ability has a cost: as the human being becomes more reflexive, he is more distant from his primary perceptive and emotional experience (Roustang, 2003). This results in a “gap” between the more primary layers of experience and the more secondarized and reflective layers of the psychic apparatus. Psychoanalytical psychotherapies are thus based on the idea that the subject may suffer from the non-represented, the non-subjectivated, and that auto-representation processes have potential therapeutic effects. It proceeds to accompany the patient toward an increased reflexivity which allows him or her to feel the not felt and to think the not thought.

We can then differentiate more precisely two main registers of reflexivity: (1) primary reflexivity concerning the relationship to emotions^17^; (2) secondary reflexivity which implies linguistic and verbal language. The most primary level of reflexivity concerns essentially how the mental apparatus manages to integrate emotions and their more “entropic” dimension. The second level relates to language and symbolic representations, which organizes the activity of thought presented by Freud as the manipulation of “small quantities of energy” by the psychic apparatus. Reflexivity thus corresponds to a heterogeneous and complex articulation of different levels of psychological functioning^18^. The “work of reflexivity” in therapy also implies that the patient speaks and that he uses language to represent his or her internal experience. But this progression through language induces a form of “subjective revolution” and a radical transformation of the relationship between oneself and others (Lacan, 1966). From the point of view of the FEP, a regulation of the free energy occurs by the mediation of language which at the same time represents and regulates the subjective experience in an original manner. As Holmes (2021) underlines “language, whether spoken, sung or gestured, is structured, ordered, negentropic” (p. 2) and language thus induces a new form of order in the psychic apparatus: we pass from an energetic order to a semiotic and signifying order. Through language, the patient “becomes aware” of certain affects and, more generally, how a way of doing things, a dream, a symptom, can be apprehended in an original and “more reflexive” way. Psychoanalytical therapies thus aim at increasing the reflexivity with the idea that this will help to patient be more in phase with the internal and the external worlds, which finally leads to a better regulation of the energetic aspects of the mind necessarily related to the FEP.

**(6) Narrativity** can be considered as a “meta” level of reflexivity. It leads to integrate the reflexive experience into a narrative framework. Reflexivity and narrativity thus maintain a close relationship because reflexivity requires a form of narrativity, an ability to “tell a story” and, conversely, narrativity favors a certain degree of reflexivity. There are different degrees of narrativity of which one of the most primary forms consists of what Bion (1965) has called the “chosen fact” -a term borrowed from the French physicist and philosopher Henri Poincaré- and which corresponds to the linking of several psychic elements (a feeling, a dream, an interpretation, etc.). The chosen fact organizes and generates heuristic emergences that give meaning to the subjective experience. They can be included into narrative scenarios or more complex “narrative envelopes” that Bion calls “vertexes” and that favor the coherence of the experience by creating a more global meaning (Rabeyron, 2018). This narrative framework is fundamental because it permits the subject to inscribe the experience in a story that represents a meta-level of organization of desires, self-esteem, relationships, anxieties, etc. (Mar and Peterson, 2013). More precisely, narrativity implies an origin, a goal, an end, scenarios, protagonists, even a spectator or an “audience,” like in a movie. The human experience would otherwise be reduced to a succession of pictures without coherence and meaning. Thus, some patients suffer from not being able to tell themselves a story, or not being able to tell certain parts of their story, when the trauma has broken into the capacity of subjective elaboration. They can no longer find meaning in their story and psychoanalytic therapies aim to increase, or “repair,” narrativity.

From the FEP perspective, as Holmes (2021) points out “the brain's aim is constantly to reduce informational entropy and maximize meaning” (p. 2). Narrativity can then be considered as the highest level of subjective experience which nevertheless must be articulated with more basic energetic aspects. In this regard, the brain can be in a “mode” called “fact free learning” or “structured learning” which aims at minimizing complexity by “compressing” representation models (Carhart-Harris and Friston, 2019): “one can refine higher-level models or narratives to make them simpler by removing redundant parameters, thereby revealing the underlying core structures and manifolds” (p. 332). This process leads to “new perspectives resulting in “aha” or “eureka” moments that “typically emerge spontaneously, 'out of the blue' as simple, elegant solutions, presumably because redundant models and/or model parameters have been unconsciously stripped away, leaving the 'bare truth beneath”' (p. 332). Thus, the psyche seems to regulate its energetic dynamics by meanings and narratives processes that are supposed to be catalyzed during psychoanalytical therapies.

## Transformational processes between chaos and order

Psychoanalytical therapies therefore require a setting (with several envelopes), a specific psychic state and several fundamental processes (transference, free association, dream, play, reflexivity and narrativity) that favor symbolization. These different elements induce effects on the subjectivity, the suffering of the patient and its symptomatic expression^19^. In this respect, several “markers” of the therapeutic effectiveness of psychoanalytical therapies can be identified^20^ (Figure 1): reduction of the symptoms, disappearance of anxieties, decline of toxic object relationships, softening of defense mechanisms and reduction of inhibitions. In comparison to therapies with predetermined goals (focused explicitly on the suppression of symptoms), psychoanalysis assumes, as Freud already proposed, that “healing comes afterwards.” The therapeutic effects will thus become manifest in a second stage, as the “apparatus for thinking the thinking,” as Bion (1965) calls it, will be transformed in depth. This is why it could be preferable not to focus explicitly on the symptomatic expression in order to achieve lasting effects on psychic reality and it may be relevant to focus on the source rather than the consequences. From the point of view of the Bayesian brain theories and the FEP, psychoanalytic therapies involve the transformation of the generative model with the idea that this will induce transformation of the subject's relationship to the internal and the external world. This is probably what Bion (1965) has tried to theorize with the “alpha function” which corresponds to the way in which the subject manages to “think” or “dream” the real, thanks to a global “psychic function” which transforms the beta elements (corporal, sensorial, emotional and non-thinkable in themselves) into alpha elements (the first “bricks” of the thought). Psychoanalytic therapies thus aim at transforming the quality of this psychic function of global integration which is necessarily, employed, in its foundations and its most basic logics, toward the need for the minimization of free energy.

The transformation of this global psychic function involves processes of great complexity and non-linear logic (Galatzer-Levy, 2016; Turtz, 2020). The interconnection of these processes is of such complexity that any attempt to separate them in order to extract empirically testable variables may lead to the destruction of the holistic character of their structure which gives them a “hyper-complex” dimension (Morin, 2005). We can therefore understand why a therapy probably cannot be carried out in all its depth in just a few sessions. A sufficient frequency and duration are needed for these processes emerge and to induce profound transformations (Leuzinger-Bohleber et al., 2019). They require time for the transference to be established and for the patient to be able to express freely about intimate experiences. The gradual establishment of a relationship of trust also takes time before allowing the patient and the clinician to be able “play” together and co-create a “narrative” of the therapy, which guarantees the coherence and the meaning of the therapeutic process.

The transformations induced by the therapy can also be envisioned as the consequence of variations of entropy and free energy. From this point of view, let us first recall that the term entropy was initially used by Clausius from the Greek êtropê (η*τρ*oπη) meaning “contained transformation.” The notion of entropy thus implies, from its origin, the idea of a transformation in a closed system. This evokes several of Bion (1970) notions and in particular this idea: “the analysis in its totality can be seen as a transformation in which an intense catastrophic explosion O has occurred at the affective level” (p. 44). This explosion can be conceived as a high level of free energy that results from a “catastrophic change” contained by the analytic setting. The processes of transformation thus seem to be catalyzed by an increase of free energy within the psychoanalytic setting which allows the containment and the “manipulation” of energy, implicating the setting and the containing dimension of the therapeutic relationship. Thus, for Holmes and Nolte (2019) “the therapeutic duo is an aid to binding potentially disruptive free energy in creative ways” (p. 6). The therapeutic relationship is thus fundamental to “containing” these entropic logics, otherwise there is a risk that they will overflow or frighten the subject's capacities of elaboration. This implies, as already mentioned, a “negative capability” (Bion, 1965), on the part of the clinician, which allows the patient to tolerate the uncertainty and the doubt which characterize states of high entropy. Both the patient and the clinician must therefore share a tolerance to uncertainty (Turtz, 2020) and a “sufficient mental space” for the development of symbolization and maturation processes.

**Figure 2:**
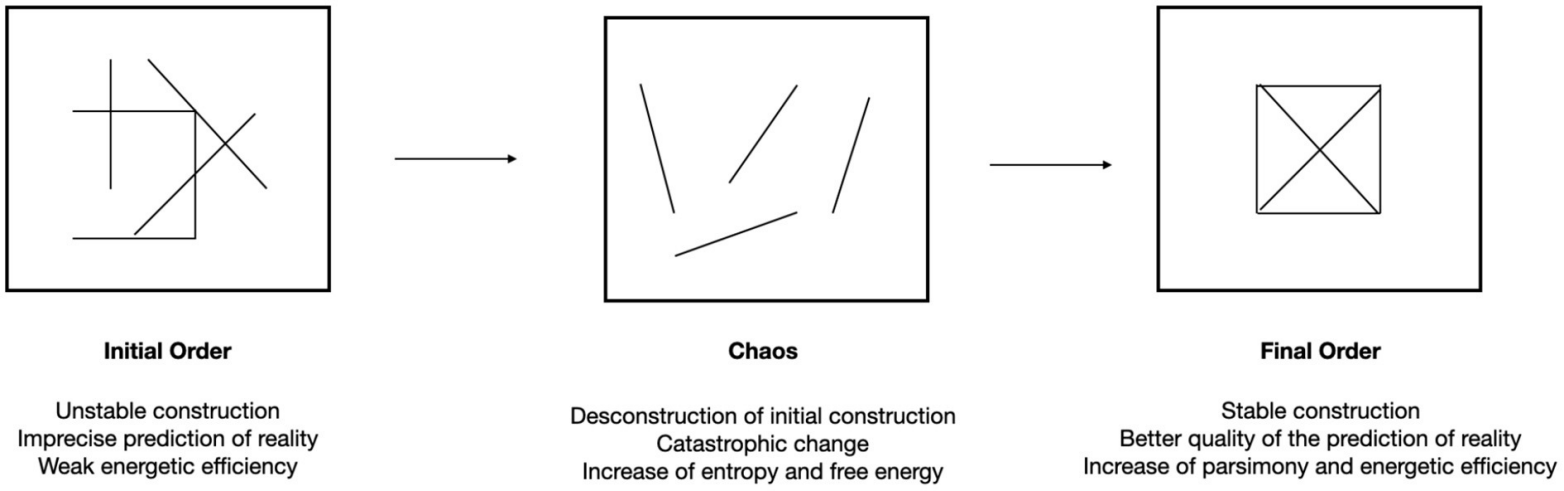
Metaphoric representation of the evolution of psychological functioning during a psychoanalytic therapy from an initial order to a final order after a more chaotic and entropic phase.

If we try to understand these entropic processes more precisely in the therapeutic setting, they seem to involve a complex interplay between an extension and a reduction of entropy, disorder and order, stochastic and deterministic processes, disinhibition and inhibition. Interestingly, this interplay between order and disorder is also reported in the neuroscience literature as noted by Cieri et al. (2021): “the fact that the brain is considered a system that wanders near a critical dynamic zone between states of order and disorder is widely accepted in cognitive neuroscience” (p. 11). This “double logic” appears to be intimately linked to the processes of transformational processes as noted by Holmes (2021): “a degree of chaos/uncertainty/free energy needs to be tolerated before new generative models can evolve. Homeostatic imprecision needs to be tolerated for a while” (p. 3). The transformation of psychic reality, and the improvement of the generative model, thus implies disorder before creating new forms of order (Figure 2).

The psychic state that characterize psychoanalytic therapies seems to favor the transition between these different states of organization. The subject can thus explore the internal experience in an original and less organized way than in the usual state of mind, as explained by Connolly (2022): “for stochastic change processes in psychotherapy, Tschacher and Haken have suggested that techniques such as mindfulness, or free association in psychoanalysis, may be examples of interventions that do not push the system state in a particular direction, but may have the effect of making a wider range of experience or behavior possible (p. 4). One of the neurobiological correlates of this state of disorganization and exploration seems to be the Default Mode Network: “It has been proposed that the Default Network has expanded its functions in support of spontaneous cognition. An entropy-extension as one of the processes of human consciousness evolution, subsequently followed by an entropy-reduction might be a specific characteristic adaptable both in phylogenetic and ontogenetic sense” (Carhart-Harris et al., 2014; Cieri et al., 2021, p. 14).

The more entropic states of psychic functioning also appear to be related to an increase of free energy at the level of emotional and primary processes and a decrease of more cognitive and secondary processes. This leads to a state of disorganization, and surprise, which must not, however, limit the subject's integration capacities, at the risk otherwise of tipping over into fright^21^ as explained by Holmes (2020) “Psychotherapy attempts to create the “duet for one” conditions where surprise becomes allowable and ultimately pleasurable (...) in the form of healing tears” (p. 45). The psychic apparatus must thus combine the need to extract and integrate new information from the internal and external world while accepting the danger of being overwhelmed while processing these new and potential disruptive information. Thus, a balance is needed between these processes of extension and reduction of free energy. We could here take as a metaphor a rice field in which the irrigation of the various plots requires a sufficient supply of water (at the risk otherwise of drying up the crops) but also a regulation of the hydraulic flow (at the risk otherwise of inducing a flood). The psychic apparatus seems to operate according to the same principle with a certain energetic “threshold” that allows to adjust these different processes adequately. Certain mental disorders can then be considered as pathologies of entropic drying (Holmes, 2020), in particular addictions, anorexia, autism, etc. (e.g., Wooldridge, 2018). Conversely, other mental disorders can be seen as pathologies of entropic excess: schizophrenia (Ciompi and Tschacher, 2021), manic episodes, ADHD, etc. (Rabeyron, 2021).

These high-entropy states can also be approached from the perspective of the randomness they induce in psychic functioning. Free association and daydreaming “soften” associative activity which makes it more random. Like a person who wanders randomly in a forest, the subject then makes discoveries while exploring internal psychic landscapes. He or she is surprised while discovering feelings, images, desires, anxieties, of which he or she did not suspect the existence and which are then the object of a subjective and reflexive appropriation. This is probably a natural and spontaneous process of the psychic apparatus which is catalyzed by the psychoanalytical setting. Indeed, human beings spontaneously goes through states of reverie as notably shown by the research on mind wandering (Mooneyham and Schooler, 2013). The psyche needs moments of release from the external world, which induces a state of disengagement from the perceptual, sensorial and motor experience because the psyche cannot, at the same time, transform itself and be in interaction with the world (Buckner et al., 2008). These transformational processes also imply a certain plasticity of the psyche as illustrated by this metallurgical metaphor proposed by Connolly (2022): “Where chaotic itinerancy is mediated through randomness, its positive effect on the functioning of the system has been formally described as simulated annealing, which is itself analogous to the process of annealing in metallurgy, where a metal is heated up over several occasions, becoming stronger in the process. By the heating process (either real or simulated such as by neural excitability), the system reaches a state of greater plasticity, allowing it to settle again, this time in a new, potentially more optimal attractor state” (p. 5). In the same manner, the psychoanalytical setting “heats” psychic reality and places the patient in a certain state of malleability.

Finally, and in passing, such a state seems to be catalyzed by psychedelics, an observation which may illuminate the nature of these processes. Psychedelics target in particular “high levels of the brain's functional hierarchy, primarily affecting the precision weighting of high-level priors or beliefs” (Carhart-Harris and Friston, 2019, p. 334). They may induce transformations through “pivotal mental states” which correspond to states of great plasticity^22^ whose fate can be transformative or pathological^23^ (Brouwer and Carhart-Harris, 2021). These states favor in-depth transformation of pathological patterns, thus allowing therapeutic impact on many different clinical symptoms. It could be a common factor with psychoanalytic therapies, given that such states favor the transformation of the structure of the psychic apparatus, which is different from psychotherapies that aim the reduction of specific symptoms. Psychedelics also induce a greater sensitivity to the environment, which also occurs in psychoanalysis when the patient regresses and projects unconscious elements onto the setting (Bleger, 1979). This underlines the fact that the internal malleability requires a malleable environment, as proposed by Winnicott (1969) with the notion transitional space and Milner (1952) with the concept of “pliable medium.” These theories suppose that processes of internal transformation require to be “projected” in the external world, and in particular on objects having specific properties. The psyche has also to be able to “synchronized” sufficiently with the environment to project these psychic contents that are then introjected in order to be symbolized. The activity of symbolization thus requires a passage through exteriority, which means that the internal malleability has to be coupled with an external malleability in order to let the processes of transformation occurring during these states of high entropy.

## Conclusion

This paper has proposed a model of the fundamental principles of psychoanalytic therapies articulated with contemporary theories of the Bayesian brain and the FEP. In this view, the main components of psychoanalytic therapies which favor transformational processes have been examined, especially the importance of the setting (with several envelopes), a particular psychic state and specific processes (transference, free association, dream, play, reflexivity, narrativity). The combination of these different elements induces after effects on the patient suffering and symptoms. Several “markers” of this therapeutic effectiveness on psychic reality have also been identified: reduction of symptoms, disappearance of anxieties, decline of toxic object relationships, softening of defense mechanisms and reduction of inhibitions.

Such a model of psychoanalytical therapies can be compared to some contemporary research in the field of cognitive neurosciences, and in particular those related to the Free Energy Principle (FEP). We can see a certain affinity between psychoanalytical metapsychology and the FEP as pointed out by Holmes (2021): “contemporary psychoanalysis psychotherapy and revitalized Freudian ideas resonate with the FEP” (p. 3). There are indeed several points of similarity between these models which help to construct a global understanding of psychic functioning taking into account the contributions of the science of subjectivity and neurosciences. This also favors the dialogue between psychoanalysis and other fields of knowledge, which puts psychoanalytic metapsychology to the test while underlining how its theoretical constructs could be useful for other disciplines, in particular the neurosciences of subjectivity.

The transformational processes induce by psychoanalytical therapies have also been analyzed by proposing several parallels with the FEP. In this regard, psychological processes seem to implicate non-linear dimensions aiming at transforming the psychic apparatus in its totality. This process of transformation takes time and requires a setting that allows the containment of high entropy processes. More precisely, these processes take place according to an interplay of extension and reduction of free energy. They create new orders from states of disorder according to a certain energetic “threshold” which allow to “update” the generative model. These states of high entropy are also characterized by their random functioning, a necessary preliminary to explore subjective experience in an original manner. States of plasticity associated with this high entropy then favor in-depth transformations of the psyche, which also requires a certain psychic malleability that has to be coupled with a sufficient malleability of the environment. The hypothesis proposed in this work nevertheless remain as a global and conceptual overview and would require much more development to be specific about the relations between psychological processes, psychotherapy and FEP.

Finally, in order to go beyond the thinking proposed in this paper, future developments could attempt to describe more accurately the links between subjectivity, consciousness, information, matter and energy. In this respect, let first recall that matter is condensed energy and that information can be considered as a certain organization of matter that requires energy. From this point of view, psychic reality can be considered as a part of matter with a highly structured organization which requires a considerable quantity of energy to be created, developed and maintained. Psychic reality is, moreover, structured in a heterogeneous way according to a fundamental divide between conscious and unconscious processes. Consciousness thus appears as a “manipulation” of information, notably in the form of representations, and is thus indirectly a form of manipulation of energy^24^. One could then consider a continuum starting from energy going the most complicated aspects of subjective experience (energy < > matter < > information < > subjectivity). It implies that energetic dimensions that organize matter -in particular the second law of thermodynamics- may have a profound, but indirect and discreet impact, on psychic functioning. What begins to take form in this way could then be a theory that would tackle the hard problem of consciousness (Solms, 2019) from the precise description of such a continuum and its potential discontinuities.

## Data availability statement

The original contributions presented in the study are included in the article/supplementary material, further inquiries can be directed to the corresponding author/s.

## Author contributions

The author confirms being the sole contributor of this work and has approved it for publication.

## Conflict of interest

The author declares that the research was conducted in the absence of any commercial or financial relationships that could be construed as a potential conflict of interest.

## Publisher's note

All claims expressed in this article are solely those of the authors and do not necessarily represent those of their affiliated organizations, or those of the publisher, the editors and the reviewers. Any product that may be evaluated in this article, or claim that may be made by its manufacturer, is not guaranteed or endorsed by the publisher.

## Footnotes

^1^The FEP is a “scale free principle”. This characteristic is important because, as Connolly and van Deventer (2017) point out, it means that the free energy principle can potentially be applied to all levels of psychic functioning. It can thus explain purely sensory and perceptual levels of information processing (e.g., visual perception) but also more complex levels of interactions pertaining to the interactions between a therapist and a clinician.

^2^whose effectiveness has been largely demonstrated by empirical research (Shedler, 2010; Fonagy, 2015; Leichsenring et al., 2015; Steinert et al., 2017).

^3^I have chosen to focus the attention on psychoanalytical psychotherapies because they seem to be particularly compatible with the theories of free energy as already shown in particular by Holmes (2020). But it might be possible to propose a more global, integrative and trans-theoretical model of psychotherapy based on the same approach.

^4^I will propose a global approach of psychoanalytical psychotherapies describing mainly what can be considered as common factors of these therapies. I cannot, in this paper, address more precisely the specificities of this approach because there are different schools of psychoanalysis and different categories of mental disorders.

^5^This topic would probably deserve a book given its complexity and I will mainly propose in this paper a global overview. The reader looking for a more in-depth analysis of psychotherapy from the point of view of FEP might look at Holmes (2020).

^6^The notion of container is used in reference to Bion's writings, insisting here on its “static” configuration, which could give the feeling that the therapist container is entirely differentiated from the patient's psychic life. This notion is described in this manner in order to insist on the containing and “solid” dimension of the setting, but actually, from a Bionian perspective, the container is a far more labile and porous notion which can also include the patient's psychic life.

^7^This envelope can be described, from a mathematical point of view, as a Markov blanket (Kirchhoff et al., 2018). A Markov blanket accounts for certain properties of Bayesian systems, and more precisely for the set of “nodes that surround a node” forming a blanket that surrounds an initial node. This leads to a differentiation between a representation of the internal states of a system and the external states of its environment.

^8^This process can take different forms in the clinic practice: the patient no longer comes to the sessions, the therapist makes a mistake, etc.

^9^Free association will be analyzed in more details in the third part of the paper.

^10^Especially emotions associated with primary processes as described by Panksepp (1998).

^11^With the fundamental difference that meditation can be practiced alone whereas psychoanalytical practices suppose that free association must be done in the presence of another person who is himself in a particular psychic state so that the processes of symbolization operate fully. Another difference is the relationship to time which is more focus on the here and now in meditation while psychoanalysis rely on a larger consideration of time (Cieri, 2022).

^12^The term symbolization requires definition. The term symbol comes from the ancient Greek *symbolon*, which derives from the verb *symballesthaï*, which means “to put together”. Originally, in ancient Greece, the symbol is more precisely a shard of pottery broken in two, each part of which is given to a contracting party, that testifies the authenticity of the contract, which can be confirmed by putting them together. In a restricted sense, the term symbolization thus designates the operation by which one thing represents another thing. In psychoanalytical models, symbolization concerns the various stages and processes that allow psychic reality to transform a “primary psychic matter” into a matter endowed with reflexivity and subjectivity. Thus, the symbolization designates how a person transforms his or her own experience in order to integrate it subjectively. We can more precisely distinguish the “primary symbolization” which corresponds to what Freud designates by the term “representation of thing”, while secondary symbolization designates what Freud calls “word representation” and rather concern the processes of psychic transformation associated with verbal language.

^13^The generative model is, in Friston's theory, the Bayesian model developed by the brain to predict the propriety of reality and whose main aim is to reduce free energy.

^14^This state is probably associated with specific neurobiological correlates as proposed by Cieri et al. (2021): “during resting state, the brain cortex prominently expresses alpha brainwaves (e.g., 10 Hz), associated with different functions, including top-down inhibitions, top-down prediction and information processing. This activity seems to be associated with the Default Network activity, as part of neural correlated of the ego integrity” (p. 13).

^15^This is probably what happens during an episode of decompensation, particularly in the field of psychosis (Ciompi and Tschacher, 2021), which results in the destruction of the “apparatus for thinking the thoughts” (Bion, 1965). The subject is, for example, invaded by the return of traumatic elements in the subjectivity but does not manage to “play” enough with these elements to integrate them. It then produces an implosion of the psychic apparatus and its models of representation leaves a deep trace in the activity of thought from which some subjects never really recover. We can therefore understand the importance for the psyche of regulating these energetic processes because they can result in a deadly danger for the psychic apparatus when the level of free energy reaches a critical level.

^16^Play involves a form of “pretending.” It is necessary to “pretend” because this creates a “gap” with the real experience, which encourages its potential integration. When the play “slips”, when we play for “real”, then we no longer play, and the risk is to see the game play overwhelmed in its function of regulation of psychic activity (Rabeyron, 2020a,b).

^17^Emotions do not emerge at a reflexive meta layer of mental functioning but rather at very primary layer of psychic life as shown in particularly by Panksepp (1998). Consequently, what is described here as a form of “emotional reflexivity” corresponds to the ability to “feel” emotions -emerging as “bottom-up” processes—that haven't been “felt” due to, for example, defense mechanisms as cleavage. So, reflexivity deals with the way that emotions will be integrated (and thus felt) at meta layer of mental functioning.

^18^Some mental disorders might be the consequence of a disintegration of these registers. For example, certain patients described as “operative” (*opératoire;* Marty, 1998) who seem to think without experiencing emotions, while conversely certain patients are overwhelmed by affects and the inability to symbolize through language expression.

^19^An analysis of the relationship between mental disorders and the FEP will not be covered in this paper because the objective is rather to study the underlying secondary processes that can lead to different forms of symptomatic expressions. A more specific analysis of the relationship between psychopathology and FEP can be found in Rabeyron (2021).

^20^Other markers could probably be identified (for example, the patient's autonomy capacities), but it seems that these markers were the most frequently reported in the psychoanalytical literature. It might also be relevant to study in more detail the relationships between these different markers, particularly from the point of view of free energy.

^21^This state of fright probably corresponds to an emergency procedure which results in the “stopping” of the processes of integration of the environment, at the risk otherwise of “exploding” the generative model as can be seen in psychosis.

^22^These states can also be induced by stress as shown also in the field of exceptional experiences (Rabeyron and Loose, 2015).

^23^At a cerebral level, three specific effects of psychedelics are observed (Brouwer and Carhart-Harris, 2021): decreased modularity, decreased hierarchy, and increased overall functionality. This gives the sense that the brain functioning is “flattened” (Carhart-Harris and Friston, 2019) resulting in a “reset” effect. In these very special moments, brain connectivity increases, modularity decreases, and brain plasticity increases. Brouwer and Carhart-Harris (2021) also show specific pharmacodynamics aspects associated with these processes with serotonin 2A receptors that are blocked by psychedelics. The more these receptors are blocked, the greater the subjective effects of the psychedelics will be. The impact on these receptors seems to participate in neuronal plasticity, which would promote long-lasting effects as well as a hyper-sensitivity to the environment when taking psychedelics.

^24^Subjective experience corresponds to a highly complex process constituted by a set of representations whose properties can be studied very precisely thanks to microphenomenology and microanalysis (Rabeyron and Finkel, 2020).
